# Open-source device for high sensitivity magnetic particle spectroscopy, relaxometry, and hysteresis loop tracing

**DOI:** 10.1063/5.0191946

**Published:** 2024-06-26

**Authors:** E. Mattingly, A. C. Barksdale, M. Śliwiak, J. Chacon-Caldera, E. E. Mason, L. L. Wald

**Affiliations:** 1 Massachusetts Institute of Technology, Health Sciences and Technology, Cambridge, Massachusetts 02139, USA; 2 Massachusetts Institute of Technology, Electrical Engineering and Computer Science, Cambidge, Massachusetts 02139, USA; 3 Martinos Center for Biomedical Imaging, Massachusetts General Hospital, Boston, Massachusetts 02129, USA

## Abstract

Magnetic nanoparticles (MNPs) are used extensively across numerous disciples, with applications including Magnetic Particle Imaging (MPI), targeted hyperthermia, deep brain stimulation, immunoassays, and thermometry. The assessment of MNPs, especially those being designed for MPI, is performed with magnetic particle spectrometers, relaxometers, loop tracers, or similar devices. Despite the many applications and the need for particle assessment, there are few consolidated resources for designing or building such a MNP assessment system. Here, we describe the design and performance of an open-source device capable of spectroscopy, relaxometry, and loop tracing. We show example measurements from the device and quantify the detection sensitivity by measuring a dilution series of Synomag-D 70 nm (from 0.5 mg Fe/ml to 7 ng Fe/ml) with a 10 mT drive field at 23.8 kHz. The device measures 260 pg Fe with SNR = 1 and 1.3 ng at SNR = 5 in spectroscopy mode in under one second of measurement time. The system has a dynamic range of 60 *μ*g to 260 pg Fe without changing the hardware configuration. As an example application, we characterize Synomag-D’s relaxation time constant for drive fields 2–18 mT and compare the magnetization responses of two commonly used MNPs.

## I. INTRODUCTION

Magnetic nanoparticles (MNPs) are utilized in a broad range of applications, including magnetic particle imaging,^1^ targeted hyperthermia,^2^ deep brain stimulation,^3^ immunoassays, and thermometry.^4^ For all these applications, an understanding of the MNP properties catalyzes effective research and development as well as improves the applications. Magnetic particle imaging (MPI) uses the unique nonlinear magnetic properties of superparamagnetic iron oxide nanoparticles (SPIONs), a type of MNP, that are driven into and out of saturation (typically with a 4–20 mT amplitude 10–50 kHz field) in concert with localizing fields to spatially map the SPION concentrations.^1^ This allows the measurement of cerebral blood volumes within the brain for neuroimaging^5–7^ or tracking CAR-T cells for cancer therapy.^8^ Recent developments in MPI tracers suggest an order of magnitude improvement may be on the horizon,^9^ but for these gains to manifest, it will require continued development of the particles with instruments that accurately reflect the magnetic fields produced in MPI. For this reason, magnetic particle spectrometers (MPSs) and magnetic particle relaxometers are often used, as they can produce the relevant field magnitudes and frequencies used in MPI but are far smaller, more cost effective, and provide more information about the SPION dynamics than full imagers. Here, we refer to the device used to record the harmonic spectra of magnetic particles’ response to an applied oscillating field, a magnetic particle spectrometer (or “spectrometer”), and the device used to measure a particle’s relaxation time constant or other related parameters, a magnetic particle relaxometer (or “relaxometer”). In MPI, the image reconstruction is greatly facilitated by having *a priori* knowledge about the SPION properties. In “System Matrix” reconstruction, a matrix is measured that maps the SPION location within an imaging space to the harmonic spectra of the received signal.^10^ Often, this measurement requires considerable imager time and a robot, though a spectrometer can be used to accurately measure the system matrix within seconds.^11,12^ For “X-Space” reconstruction,^13^ the SPION relaxation time constant should be estimated and is typically derived from a relaxometer measurement.^14–16^ Spectrometers can also inform the synthesis process for SPIONs.^17^

Magnetic particle spectroscopy can also be a valuable scientific instrument on its own. Draack *et al.* demonstrate that spectroscopy measurements have a notable temperature dependence, and Bui *et al.* have further shown this can be useful for remote thermometry.^18,19^ It can also be utilized for the detection of viruses with immunoassays, among others.^4^

In addition to MPI, SPIONs are used as a targeted hyperthermia agent by driving the SPIONs with higher amplitude fields and at higher frequencies. The heating can be clinically used for cancer therapies by directly heating malignant tissue surrounding the SPIONs to trigger apoptosis or by facilitating targeted drug delivery.^20^ Efficacious therapy requires an accurate model of the applied thermal dose, which is intrinsically coupled to the shape of the dynamic magnetization vs external field [m(H)] curve. Therefore, benchtop characterization of the SPION m(H) characteristics with a hysteresis loop tracer is critical for the refinement of the synthesis processes.^2^

Other magnetic characterization devices exist, such as vibrating sample magnetometry (VSM)^21^ and superconducting quantum interference device (SQUID) magnetometers.^22^ Neither is ideal for the characterization of MPI agents, as VSM is not tailored to the measurement of the dynamic magnetization at 10 kHz to 1 MHz, and SQUID introduces considerable complexity and cost (e.g., a cryogenic sensor) but is able to measure these dynamics.^23,24^ AC-susceptometry devices are more similar in function, yet they do not typically measure the exact harmonic spectra but rather quantify the complex susceptibility of the sample, which is a linear characteristic.

Numerous spectroscopy, relaxometry, and hysteresis loop tracing devices have been built,^11,12,14–18,25–31^ each with its own focus. Early magnetic particle spectrometers and relaxometers sought to elucidate particle parameters such as size distribution^27^ or measure the time constant of commercial SPIONS,^16^ though literature of specific hardware developments was reported not long after, such as using eddy-current shielded enclosures for filter components^2^ and low inductance drive coils in tandem with ballast resistors for wide bandwidth SPION measurements,^15^ application specific preamplifiers and receive coils,^32–34^ and noise matching.^35^ Improvements in the receiving electronics and specialized drive coils facilitated the first system matrix measurement in 2D^12^ and then in 3D.^11,36,37^ Hardware was also developed for making quasistatic approximations of the SPIONs at various bias fields,^28^ as well as custom coils to accommodate very large sample volumes to track the growth of SPIONs.^17,29^ Others have developed low-cost signal generation platforms that could be used for these applications,^38^ as well as signal calibration techniques.^39^ Each of these developments is significant in its own domain, but there are few consolidated resources enabling the reproduction of those devices. There is also minimal literature on combining relaxometry, spectroscopy, and other similar techniques into a single device capable of high-sensitivity analysis of MNPs.

**FIG. 1.**
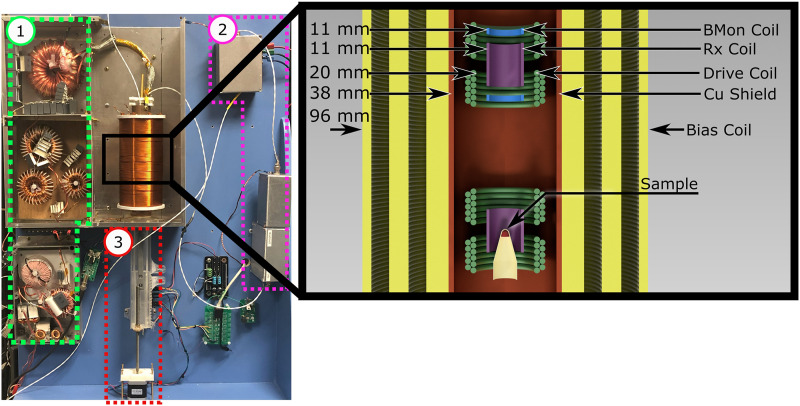
Photograph of the system. The size of the benchtop device is about 80 cm tall by 60 cm wide. No. 1, green: The drive filter is where the raw signal from the amplifier comes from, and there are three separate shielded regions to prevent cross-talk. The topmost toroid is the current transformer, which connects to the drive coil. No. 2, purple: The receive signal goes to the enclosures on the right-hand side of the photograph, where it is filtered and scaled by a transformer before being amplified by the preamplifier at the top. No. 3, red: The sample positioner and motor. This photograph excludes the data acquisition hardware, bias amplifier, and power supplies for the preamp and stepper motor. The illustration on the right shows a cross-section view of the coils. In yellow are the bias coils; in copper is the shielding tube; in green are the drive coils; in purple are the receive coils; and in blue are the B-field monitor coils.

Here, we present an open-source device for the assessment of MNPs and the associated design process, lowering the cost and investment in engineering needed to reproduce this laboratory tool. Figure 1 shows a photograph of the system as well as an illustration of the coil arrangement.

There are three main coils in the device: a receive coil, a drive coil, and a bias coil. Each is described in detail in Sec. III A. In brief, the drive coil (sometimes called the transmit coil) produces a 10 mT peak magnetic field at about 23.8 kHz to drive the MNPs magnetization in and out of saturation. The receive coil inductively senses this changing magnetization via Faraday detection. The bias field slowly (∼10 Hz) changes the quasistatic operating point of the MNPs magnetization.

We characterize the performance of the device in five functional modes: (1) MNP spectroscopy—to accurately measure their spectral magnetization response, (2) Relaxometry—to determine their relaxation time constant, (3) Hysteresis loop tracing—to plot their hysteresis loop, (4) Magnetometry—to measure their quasistatic magnetization curve, and (5) 1D system matrix measurement—for use in MPI image reconstruction.

## II. DESIGN GOALS

### A. Sensitivity goal

Many applications of the spectrometer require a sensitivity equal to or greater than the MPI systems. For example, validation of a sample’s Fe mass for possible deterioration before it is injected or checking dilution series linearity. Therefore, the device should be equal to or more sensitive than the state-of-the-art MPI systems. Previously published imagers have demonstrated detection limits ranging from about 1 ng Fe to 100 ng Fe.^34,40–42^ This suggests a sensitivity goal of ∼5 ng of Synomag-D or less should be detectable with at least a signal-to-noise ratio (SNR) of 5 within 5 s of acquisition time.

### B. Device and sample size goal

The device is designed to fit comfortably on a lab benchtop or be wall-mounted to minimize its footprint and accommodates a standard sample container, the 0.2 ml microcentrifuge tubes (∼6 mm outer diameter), as they are also a standard sample holder for imagers (see Ref. 43). Furthermore, this size will also accommodate many other standard lab nuclear magnetic resonance (NMR) bulbs or tubes.

### C. Drive field amplitude, stability, purity goals

The drive coil is responsible for creating an oscillating magnetic field applied to the sample, driving the sample’s magnetization into and out of saturation (typically). To sufficiently saturate most MNPs commonly used in MPI, a drive field of about 10 mT peak is needed. While a higher drive field amplitude likely increases sensitivity, many MPI systems operate around 10 mT, though there are many exceptions, both higher and lower. It is essential that this waveform is harmonically pure and temporally stable (magnitude and phase), as the drive field is inherently coupled to the signal source (the sample’s magnetization). Noise or drift in the drive field will be reflected as noise or drift in the sample signal.

The stability goal for the drive field is a magnitude drift of 0.1% per 100 measurements (each about 80 ms long) and 1 milliradian per 100 measurements of phase drift. These values were chosen because this amount of drift can easily be subtracted off in post-processing.

To estimate the harmonic purity necessary, we suppose the amplitude of the detected third harmonic of the drive field should be at most the same order of magnitude as a sample of 10 ng Fe (roughly sensitivity goal).

The equivalent magnetic moment of the “feedthrough” drive field is

$$M_{d}=N\cdot I\cdot A\cdot A_{\mathrm{Grad}},$$ (1)

where N · I is the amp-turns of the drive coil (∼250 Amp-turns), A is the cross-sectional area (∼3 ·10^−4^ m^2^), and A_Grad_ is the “gradiometer” attenuation (approximated by −60 dB for MPI gradiometers^40,44^), so M_d_ is roughly 7.5 · 10^−5^ Am^2^. The third harmonic component of this should be at most a similar order of magnitude to 10 ng Fe’s third harmonic component, whose magnetic moment can be expressed as

$$M_{Fe}=m_{Fe}\cdot M_{\mathrm{Sat}}\cdot S_{3/1},$$ (2)

where m_Fe_ is the iron mass (kg), M_Sat_ is the saturation magnetization per mass (∼110 Am^2^ kg^−1^), and *S*_3/1_ is the ratio of the third harmonic to the fundamental signal component (which depends on the SPION’s magnetization curve but is often about 1/3). From this, the ratio of 3f_0_ to f_0_ of the drive field (*D*_3/1_) can be expressed as

$$D_{3/1}=\frac{m_{Fe}\cdot M_{\mathrm{Sat}}\cdot S_{3/1}}{N\cdot I\cdot A\cdot A_{\mathrm{Grad}}},$$ (3)

is roughly 5 · 10^−6^, or −106 dBc.

### D. Bias field amplitude goal

Ideally, the maximum strength of the bias field would be significantly higher than the MNP’s saturation magnetization, thereby allowing the device to probe the entirety of the magnetization curve. We target 50 mT to saturate Feraheme (AMAG Pharmaceuticals, Waltham, MA, USA), a clinically approved iron oxide agent, as well as most MNPs used in MPI.

## III. METHODS

### A. Hardware design

**FIG. 2.**
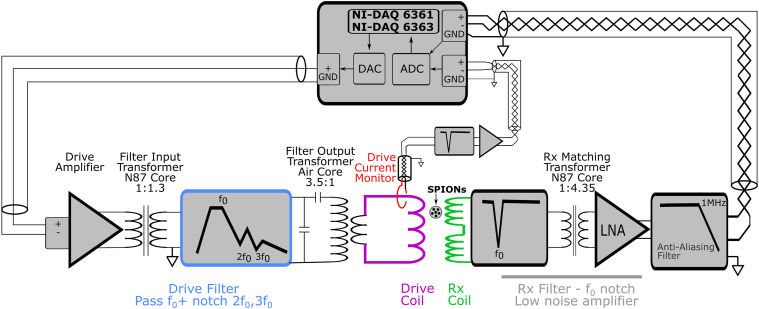
Overview of the electrical systems. Not included in this figure is the bias coil system or the field sense coil.

The spectrometer primarily consists of (i) the drive assembly, (ii) the receive assembly, (iii) the bias assembly, and (iv) the sample positioner. The overall electrical layout of the drive and receive assemblies is seen in Fig. 2, and the coils are specified in Tables I.

#### 1. Drive coil

The drive coil itself consists of two identical subsections, where each subsection drives the field over each half of the receive (Rx) gradiometer. Each subsection is designed to produce about 10 mT at 23.8 kHz. To reduce design complexity, only passive air cooling is used.

##### a. Drive coil design.

**FIG. 3.**
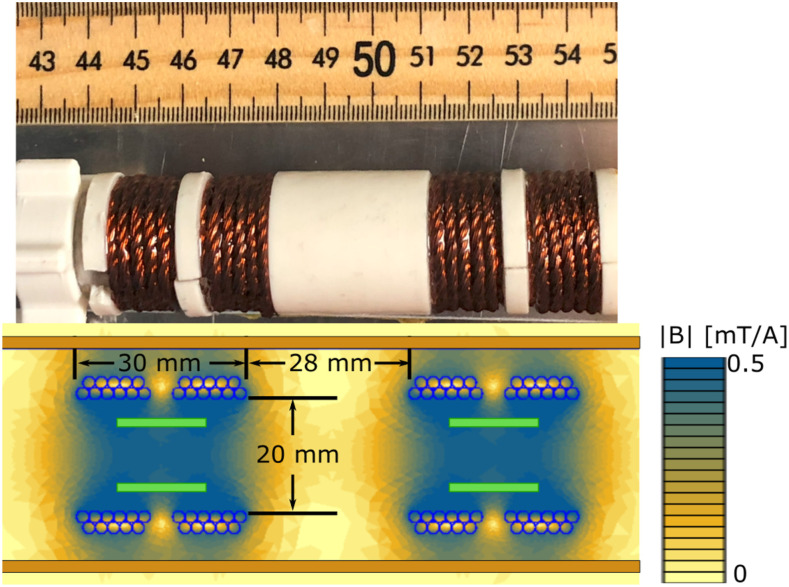
Top: Photograph of the drive coil outside of the shielding tube. Bottom: Simulated field per ampere in the drive coil at 23.8 kHz. The pairs of horizontal green lines indicate the location of the receive coils and the copper.

Figure 3 shows a photograph of the drive coil design as well as the simulated fields. As illustrated in Fig. 3 bottom, the wires were arranged in a Helmholtz-like configuration where there is a 4 mm gap in the center (axially) to improve homogeneity within the volume of the receive coil. Outside of this center gap, two layers of turns were wound, with a total of 12 turns on the first layer and 10 turns tightly wound in the grooves of the first. Adding more turns axially (i.e., elongating the solenoid) does not substantially increase the “coil sensitivity” (field per Amp) as the turns are quite far from the region of interest. More turns with larger diameters, such as additional layers, also do not improve the efficiency much as they necessarily lie at an increased radius. The increased radius simultaneously increases coupling to the shielding tube and increases the distance to the region of interest. For this design, 22 Amps are required to achieve the 10 mT goal, which is a current density of about 15 A/mm^2^. In general, passive air cooling can effectively dissipate the heat from only a 5 A/mm^2^ current density in copper applied at 100% duty cycle. However, our desired coil geometry (winding area) requires a current density of 15 A/mm^2^ to achieve the drive field goal of a 10 mT peak. This necessitates lowering the duty cycle of the drive current. Because power scales with the square of the current, an 11% duty cycle is required for the same power dissipation as 5 A/mm^2^. In normal operation, the system is run at a duty cycle closer to 30% during acquisitions and takes advantage of the time required to load and move samples as “cooling breaks” to avoid heat buildup.

Operating at 100% duty cycle would be an excessive amount of heating, and as such, the duty cycle is kept lower (∼30%) to prevent overheating.

##### b. Shielding tube.

The copper tube around the drive coil (copper colored in Fig. 1 cross-section) serves to shield the receive coil from external interference. The inner diameter of the tube is 37 mm, and it is 1.8 mm thick. The thickness was picked to be far greater than the skin depth of copper at 23.8 kHz (∼400 *µ*m). The diameter was picked to balance the detrimental effects of the shield on drive and bias coil efficiencies. While a larger diameter shield increases drive field efficiency, it lowers bias field generation efficiency since the bias field coil is placed outside the shield. The field at the center of a finite-length solenoid within a long, shielding tube multiple skin-depths thick is approximated^45^ as

$$B{|}_{\mathrm{Center}}=\frac{\mu_{0}NI}{L}\cdot \left(1-\frac{r_{\mathrm{Solenoid}}^{2}}{r_{\mathrm{Shield}}^{2}}\right).$$ (4)

Therefore, a shield diameter of 37 mm and a mean winding diameter of 24 mm reduce the drive efficiency (field per amp) by ∼40%, a tolerable reduction. The bias coil efficiency is also affected by the shield radius. While a larger shield radius increases drive coil efficiency, it also reduces bias coil efficiency since it puts that coil farther from the sample and also increases its resistance and inductance.

##### c. Wire.

The drive coil uses custom Litz wire (18/28 AWG Litz) of insulated 28 AWG (0.36 mm) strands, but no insulating shell around the bundle to facilitate epoxy stabilization of the individual strands. The skin depth at 23.8 kHz is 0.42 mm, so the current penetrates the full depth of the wire. Larger strands would yield higher AC resistance, and smaller strands would be unnecessary effort and more fragile. Larger wire bundle diameters (i.e., more strands in parallel) would limit the coil to one layer, and turn density would be sacrificed, complicating impedance matching and winding uniformity.

##### d. Coil former.

The wires were wound directly on a borosilicate glass tube with 3D printed (Formlabs 3L, Somerville, MA) spacers. Glass was picked for its low coefficient of thermal expansion.

##### e. Electromagnetic simulation.

FEMM 4.2^46^ was used to simulate the fields and impedance of the drive coil. For the simulation, the drive coil wires were specified as 18 strands of 28 AWG Litz wire, and the copper tubing was the default copper material. The simulation was run at 23.815 kHz. The simulated inductance was 8.13 *μ*H with a resistance of 62.4 mΩ. The measured inductance was 8.33 *μ*H with a resistance of 57.5 mΩ.

##### f. Current transformer.

An air-core current transformer couples the drive filter to the drive coil to (i) provide common-mode isolation between the filter and coil, thus preventing a possible source of interference, (ii) transform the coil’s impedance from (0.057 + 1.24j Ω), which would require large (∼10μF) capacitors to match, which were less available, and (iii) lower the current in the matching capacitors and filter compared to the drive coil (where the current is necessary) to reduce thermal drift. The air-core toroidal transformer transformer is modeled with the “cantilever model” for transformers^47^ with a leakage inductance of 46.5 *μ*H, a magnetizing inductance of 88.9 *μ*H, and an inductance ratio of 1:0.082 (turns ratio = 1:0.286). Air cores were used for their linearity when compared to ferrous cores. The transformer’s secondary (coil-side) wire is 5 × 3 × 28/36 AWG Litz wire. It needed to have substantially larger wire than the drive coil and contain few turns to minimize the resistance of the transformer. The primary-side wire is 115/36 AWG, but the losses are less impactful on the primary side because the current is far lower on the primary than the secondary. The effective load of the drive coil/transformer when measured from the primary-side of the transformer is 14.0j Ω (93.9 *μ*H) + 420 mΩ.

##### g. Drive filter.

**FIG. 4.**
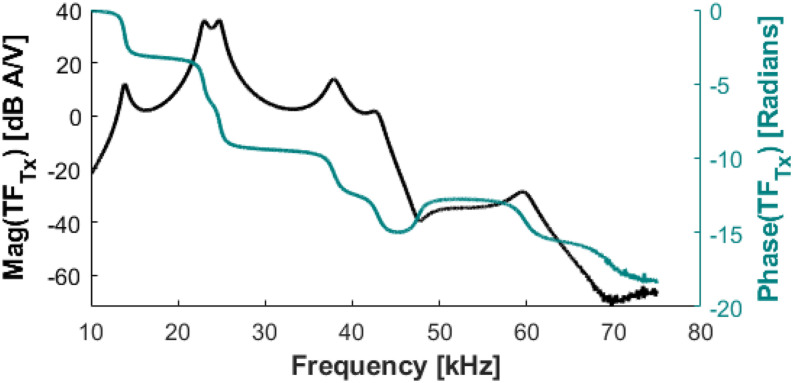
The measured transfer function between the input voltage to the drive amplifier and the filter and output current in the drive coil (transconductance) referred to as the TF_Tx_ plot. Reproduced with permission from E. Mattingly, “Design, construction, and validation of magnetic particle imaging systems for rodent, primate, and human functional neuroimaging,” Ph.D. thesis, Massachusetts Institute of Technology, 2024.^48^

The design of the filter generally follows Ref. 43, with the main modification being an output transformer. Figure 4 shows the measured filter response (amps in the drive coil per volt input to the drive amplifier).

The filter’s input transformer is designed on an N87 (R 63.0 × 38.0 × 25.0, A_e_ = 306 mm^2^, L_e_ = 152 mm, TDK Lambda, Tokyo, Japan), and to prevent additional harmonics being produced in the transformer, the flux is kept to 10 mT in the core (under 5% saturation). The core flux is expressed as

$$B_{\mathrm{peak}}=\frac{V_{Pk}}{2\pi fNA_{e}}.$$ (5)

With a peak primary voltage of 20 V (to give ∼10 mT), a frequency of 23.8 kHz, and a peak transformer core flux density of 10 mT (below 5% saturation), 40 turns are required on the primary side. The implemented transformer was also modeled with the cantilever model, which showed a leakage inductance of 7.36 *μ*H, a magnetizing inductance of 8.51 mH, and an inductance ratio of 1:1.708 (turns ratio = 1:1.30).

##### h. Drive current measurement.

The drive coil current is sensed with a Rogowski coil^49^ (15 mm inner diameter, 30 mm outer diameter, 32 turns) placed on a wire leading directly to the drive coil (i.e., on the secondary side of the air core transformer). Because detecting distortions on the order of 10 ppm is not feasible with 16 bit digitization (the least-significant bit is about 15 ppm) without further processing such as over-sampling and averaging, a notch filter (a second order LC band-stop with a measured 40 dB notch depth) is used to reduce the dynamic range. After filtering, the signal is amplified by a battery powered low-noise instrumentation amplifier (INA217, gain = 201, Texas Instruments, Dallas, TX, USA). After digitization, the signal is corrected for the effect of the filter response.

##### i. Drive field measurement.

A two-turn coil that surrounds the compensation side of the receive coil (blue in Fig. 1) monitors the drive field (“BMon”) to provide complementary information to the Rogowski coil because the field is not in phase with the current due to the phase-shifting from eddy currents in the tube. The BMon coil is only two turns (one on each side of the Rx), and each turn is the same handedness. The signal is directly digitized by the data acquisition (DAQ) as a differential measurement.

#### 2. Bias coil

The bias coil alters the MNP’s operating point on its m(H) curve by adding a near-DC offset to the sinusoidal drive field, thereby allowing the drive coil to excite a different region of its magnetization curve. This can also be used to simulate the MNP’s in MPI being moved to different axial locations.

This biasing is performed at a low frequency (11.9 Hz) to minimize the effect of the $\frac{dB}{dt}$ from the bias on the received signal. By biasing slowly, the bias coil’s inductive impedance is also minimized, therefore allowing more of an amplifier’s available power to go toward the real part of the load. The audio amplifier (RCF IPS 400, RCF S.p.A.CF, Reggio Emilia, Italy) utilized is an AC-coupled, class A/B amplifier with a cutoff ∼10–20 Hz. The bias-coil has a total of 1870 turns in three groupings with 6 mm gaps between every group, as seen in Fig. 1. The gaps allow for airflow and effective cooling. The innermost grouping consists of 5 layers of 130 turns of 16 AWG wire. The middle group consists of 2 layers of 130 turns each with 16 AWG and 3 layers each with 160 turns of 18 AWG. The 18 AWG turns were wound directly on top of the 16 AWG turns. The final grouping has 3 layers of 18 AWG, where each layer has 160 turns.

#### 3. Low-noise amplifier and receive coil

##### a. Receive coil.

**FIG. 5.**
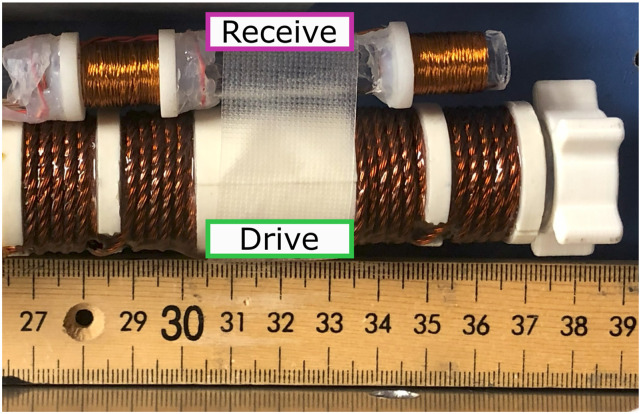
Photograph of a transmit and receive coil, side-by-side. In operations, the receive is coaxial and centered with the drive to minimize the amount of feedthrough.

The receive coil (photographed next to the drive coil in Fig. 5) is a two-part gradiometer, where each half is wound with opposite handedness relative to the drive coil, so the induced voltage from the drive coil has an opposite sign. This (ideally) nulls the net voltage across the full receive coil, leaving only voltages due to the sample magnetization.

The Rx coil is wound on a borosilicate glass tube to prevent thermal expansion if the bore warms with the drive coil. The inner diameter is 9 mm to fit the sample with a 1.5 mm clearance radially.

Each half is a 50-turn solenoid with a length of 14 mm and a winding inner diameter of 11 mm. The length/diameter was designed to be roughly 1.5 to balance coil sensitivity and homogeneity (longer coils are more homogeneous but less sensitive). The turn count was picked by making the wire-length of the receive coil roughly equal to the drive coil, potentially mitigating wire-length effects.^50^ This also keeps the surface area low, limiting parasitic capacitance generated by feedthrough of the drive waveform. By keeping the turn count fairly low, the inductance and self-capacitance are also kept low, mitigating the risk of self-resonance.

**FIG. 6.**
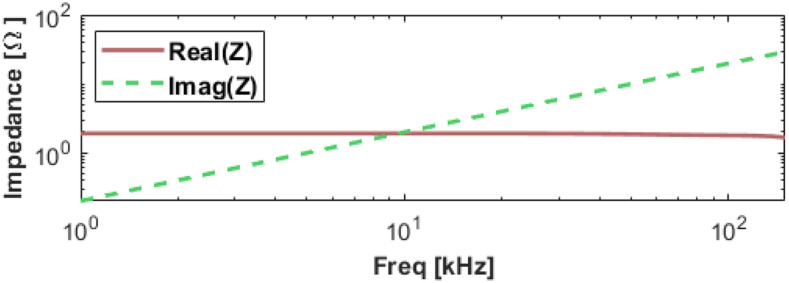
Real and imaginary parts of the receive coil impedance. The coil resistance is 1.79 Ω, and the inductance is 31.4 *µ*H. Reproduced with permission from E. Mattingly, “Design, construction, and validation of magnetic particle imaging systems for rodent, primate, and human functional neuroimaging,” Ph.D. thesis, Massachusetts Institute of Technology, 2024.^48^

The receive coil’s impedance was measured with an in-house device (available at: “github.com/EliMattingly22/Simple_Impedance_Analyzer”) from 1 to 150 kHz. The impedance plot is seen in Fig. 6. Those data show the impedance has a nearly constant real component from 1 kHz up to about 150 kHz, indicating the Litz wire mitigates the skin and proximity effects up to 150 kHz.

##### b. Low-noise amplifier.

The low noise amplifier (“LNA” or “preamp”) was designed to amplify the signal such that downstream effects do not add significant noise, and the dynamic range of the ADC is utilized.

Noise matching for inductive loads has been previously discussed in Ref. 35. We utilized the ADA8429 (Analog Devices, Wilmington, MA, USA). There should theoretically be about 1.5 $pA/\sqrt{Hz}$ of current noise and $∼3.3\;nV/\sqrt{Hz}$ of input voltage noise with a gain of 20. To match this preamp, the load impedance should be ∼2200 Ω. If feedthrough cancellation techniques are utilized (e.g., active cancellation^44^) or steeper notch filters are used, higher gains can be achieved with better noise performance.

##### c. Matching transformer.

Because the impedance presented to the preamp by the receive filter is much higher than the ideal noise matching impedance, a matching transformer is used. The voltage across the transformer’s primary is quite low, and an increased signal distortion has not been observed. The transformer is a ferrite toroid (4C65, TX 36 × 23 × 15 mm, Ferroxcube, New Taipei City, Taiwan) with an inductance ratio (coil side:preamp side) of 1:18.9 (turns ratio of 1:4.35). It also has the benefit of reducing the common-mode noise that may be present, although a fully balanced filter would be necessary to more thoroughly reduce common-mode noise susceptibility.

Referring the preamp noise to the primary side (Rx coil side) of the transformer gives about 6.5 $pA/\sqrt{Hz}$ of current noise and $∼0.76\;nV/\sqrt{Hz}$ of voltage noise.

##### d. Receive filter.

**FIG. 7.**
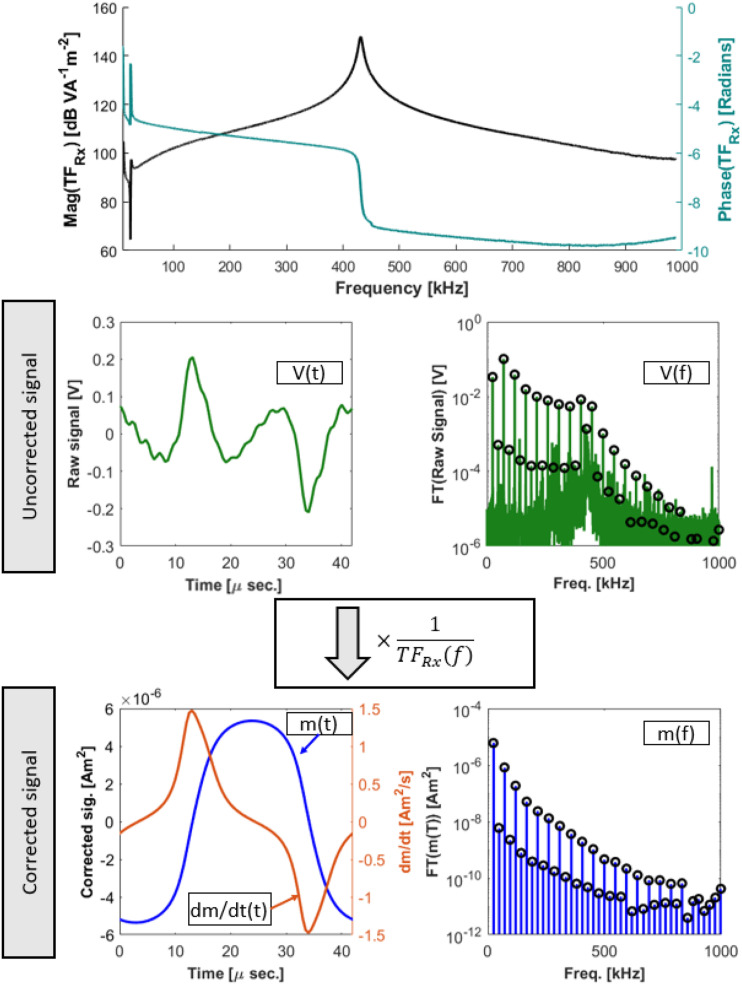
Top: The receive filter transfer function (notch depth ≈30 dB), from magnetic moment in the Rx coil to voltage out of the preamp with the notch and transformer in place. Middle left: The raw received signal for 60 *μ*g Fe. Middle right: The Fourier transform of the received signal before any transfer function correction is performed. Black circles represent the signal at each of the harmonics of the drive frequency. Bottom left: The blue trace shows the time-domain signal after transfer function correction and after all of the non-harmonic values have been zeroed. The orange trace shows its time-derivative. Bottom right: The Fourier transform of the transfer function corrected the data [m(t), not dm/dt]. Reproduced with permission from E. Mattingly, “Design, construction, and validation of magnetic particle imaging systems for rodent, primate, and human functional neuroimaging,” Ph.D. thesis, Massachusetts Institute of Technology, 2024.^48^

**FIG. 8.**
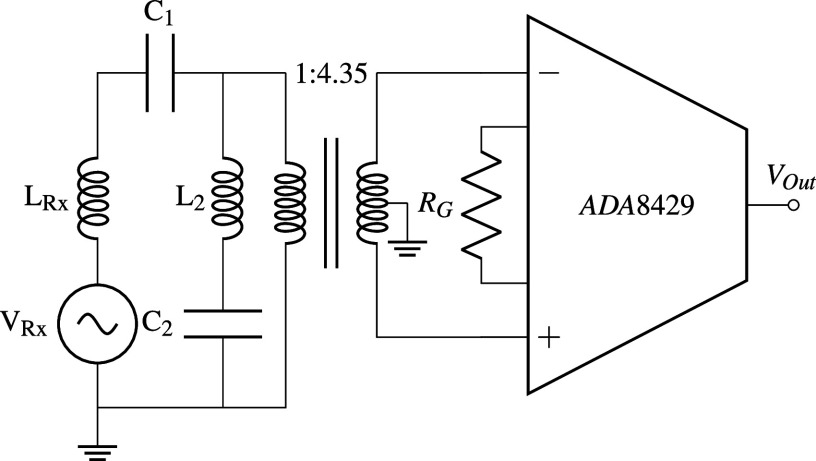
Receive path circuit model. From left-to-right: signal voltage source and receive coil (LRx), notch filter C_1_ = 915 nF, L_2_ = 158 *µ*H, C_2_ = 278.4 nF, the transformer, which scales the effective impedance of the Rx coil and simultaneously mitigates common-mode noises and gives a path for bias currents to flow (turns-ratio of 1:4.35), and the preamplifier (ADA8429) with R_G_ = 330 Ω. An antialiasing filter is needed after the preamplifier, but you can use a commercial active or passive low-pass filter.

A weak notch filter is used to reduce the amplitude of the drive feedthrough, and the filter response is seen in Fig. 7. Figure 8 shows the circuit, including the Rx coil and preamp. The shunt inductor (L_2_) was designed to be larger than the receive coil inductance to prevent voltage division between the two in the pass-band.

The filter balances the signal amplitude between the different signal bands and enables higher gain on the preamp, thus lower noise at the harmonics. Additionally, because the DAQ has only 16 bits, only a dynamic range of 10^5^ can be measured at best. By having less gain at higher amplitude signals, the signal dynamic range is compressed, allowing more harmonics to be measured.

For applications that focus on the detection of fundamental or very large samples, the filter and impedance matching transformers could be bypassed, though this has not been performed for any data presented here. For example, when using magnetometry mode, the notch is detrimental, and disconnecting it would improve the signal fidelity.

#### 4. Automatic sample positioner

**FIG. 9.**
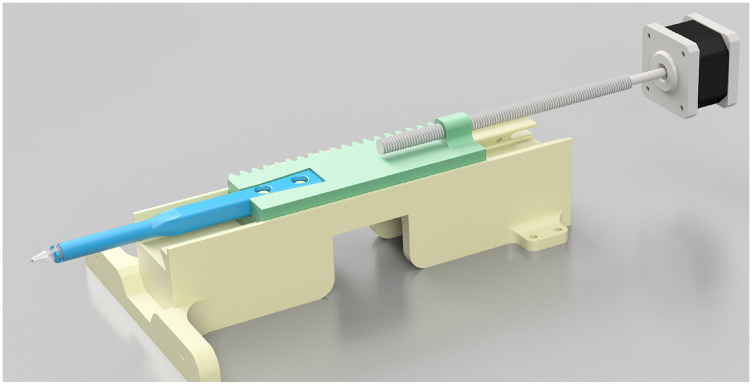
Digital rendering of the sample positioner assembly. The sample-arm is illustrated in blue. This part can be replaced for different sample geometries and is screwed into the sample car in green. The beige component is the bed rail, which has the mating dovetail groove that the bed moves on. Reproduced with permission from E. Mattingly, “Design, construction, and validation of magnetic particle imaging systems for rodent, primate, and human functional neuroimaging,” Ph.D. thesis, Massachusetts Institute of Technology, 2024.^48^

Figure 9 shows the sample positioner assembly. The sample is held by a (∼130 mm) long cantilever sample-arm screwed into a moving sample car. The sample car is driven by a stepper motor and has lead-screw and sides on a built-in dovetail, which mates with a complimentary groove on the bed rail. This design allows for easily interchangeable sample arms for different sample types (e.g., microcentrifuge tubes, NMR bulbs, etc.). In addition, a homing micro-switch on the stationary rails indices the sample’s position before each measurement.

This apparatus allows for the sample to be rapidly removed and re-inserted accurately. For instance, to measure samples with very small quantities of iron, it is useful to compare the sample-in signal to the sample-out signal repeatedly to enable statistical confidence in the measurement.

### B. Software and data analysis

#### 1. Basic operations

The system is programmed with LabVIEW 2021 (National Instruments, Austin, TX, USA) to facilitate accurate synchronization, a simple-to-use graphical interface, and ease of development. All data acquisition was performed with an NI-DAQ-6361, and the analog output signal was performed with an NI-DAQ-6363. While we used two for convenience, a single DAQ could perform both the data acquisition and analog output signals (Table II).

**TABLE I.** Measured coil parameters for the drive, receive, and bias coils.

Parameter	Value
Drive coil sensitivity (mT/Amp)	0.5
Drive coil inductance (*μ*H, 23.8 kHz)	8.3
Drive coil resistance (mΩ, 23.8 kHz)	57
Drive coil turns, per half	22
Drive coil axial length, per half (mm)	30
Bias coil sensitivity (mT/Amp)	9.36
Bias coil inductance (mH, 10 Hz)	72.7
Bias coil resistance (Ω, 10 Hz)	8.89
Bias coil turns	1870
Bias coil axial length (mm)	185
Receive coil inductance (*μ*H, 100 kHz)	31.4
Receive coil resistance (Ω, 100 kHz)	1.79
Receive coil turns, per half	50
Receive coil axial length, per half (mm)	14

**TABLE II.** System parameters for normal operation.

Parameter	Value
Sampling rate (MHz)	2
Drive period (freq.) [*μ*s (kHz)]	42.0(23.8)
Bias period (freq.) [ms (Hz)]	84(11.9)
Drive periods per bias period	2000

For the receive and transmission, the timing is triggered internally to maintain consistent timing, and the transmission frequency of the drive coil is picked to be a multiple of the sampling rate, so every period has the same number of samples and each period is identical. This prevents timing errors from propagating. When receiving, either the receive coil is sampled or the current monitors, never both simultaneously. This is because the inputs are multiplexed, and interleaving causes slight capacitive coupling between channels, causing a “ghosting” effect and potentially confounding the data. This could also be mitigated by using separate data acquisition modules for different channels, but the software was written assuming only one DAQ to limit necessary hardware costs.

#### 2. Data pre-processing

**FIG. 10.**
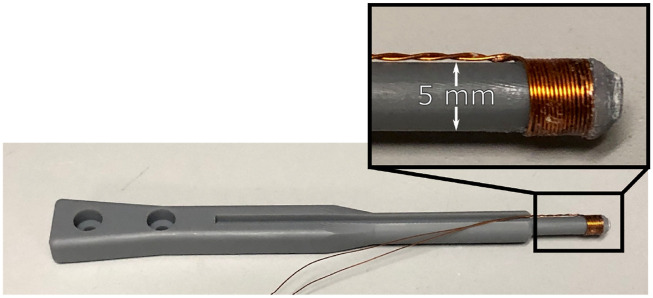
Calibration coil photograph. The coil is inserted within the Rx coil to measure the receive path transfer function. Wire is 28 AWG. Reproduced with permission from E. Mattingly, “Design, construction, and validation of magnetic particle imaging systems for rodent, primate, and human functional neuroimaging,” Ph.D. thesis, Massachusetts Institute of Technology, 2024.^48^

Regardless of which acquisition scheme is being performed (e.g., spectroscopy, magnetometry), the data first have a baseline subtracted from it. This baseline is acquired with an identical pulse sequence with the sample out of the bore, and the Rx signal and BMon are saved (uncompressed time domain). After the baseline is subtracted, a transfer function correction is performed to account for the frequency response of the Rx filter and amplifier.^39^ The frequency response is measured by using a test coil (15 turns, diameter = 5 mm, seen in Fig. 10), placed in the center of the Rx coil, sweeping different frequency waveforms across it, and then simultaneously measuring the current in the coil and the output voltage from the Rx coil. Then the response is

$$TF_{Rx}\left(f\right)=\frac{V_{Rx}\left(f\right)}{N\cdot I\cdot A},$$ (6)

where TF_Rx_ is the transfer function (units = Volts per Am^2^) that relates a magnetic moment to output voltage. The correction is applied by taking a Fourier Transform (FT) of the time-domain voltage and dividing the one-sided FT by TF_Rx_. The corrected FT is concatenated with its complex conjugate so that conjugate symmetry is conserved and the inverse-FT is real-valued. Therefore, where the raw time-domain signal is a voltage at the output of the preamp, the transfer function correction converts this to a magnetic moment (not its time derivative). Figure 7 shows an example of this process as well as the measured transfer function used.

#### 3. Spectroscopy

For spectroscopy, typically only the drive coils are enabled (the bias coils are not powered), as seen in Table III. This drives the MNPs into and out of saturation. The induced Rx signal is the temporal derivative of the magnetization response and will contain both the fundamental drive frequency and its harmonics. These harmonics are of particular interest in MPI (e.g., the 3f_0_ to 5f_0_ ratio^51,52^), as they are unique to MNPs. A single spectroscopy “measurement” applies 500–2000 periods of the drive field (20–80 ms) twice. In the first application, the ADC records the Rx signal. The second application records the drive current monitor signal. A stepper motor connected to the sample holder via a lead-screw allows automated movement of the sample in and out of the receive coil. Eleven measurements are made with the sample outside the Rx coil, followed by 11 measurements with the sample in the coil. This cycle is repeated 2.5 times. The sample-out measurement time-series is analyzed to provide a linear model of the baseline drift, which is then removed from the sample-in time series. The pseudo-code for this processing is included in the Appendix. In brief, the magnetization data are Fourier transformed, and each harmonic is as follows:

1. Separated into sample-in vs sample-out data and smoothed with a five-point rolling average.
2. A model of complex signal drift is calculated from the linear fit between the mean signal of the first and last five sample-out measurements.
3. The signal drift model is interpolated to approximate the expected baseline drift, which is then subtracted from the data.
4. The signal drift model is interpolated to approximate the expected baseline drift, which is then subtracted from the data.
5. The signal amplitude could then be determined from a simple subtraction of the mean sample-in vs sample-out data. Alternatively, we analyze the time series using a general linear model (GLM) consisting of a constant term, a linear term, and a binary function representing the sample-in vs sample-out levels (the signal level regressor) and determine the signal level from the amplitude of the sample-in vs sample-out regressor. The noise level is taken as the standard deviation of the residual signal of the sample-out measurements after fitting the GLM.

**TABLE III.** The typical acquisition parameters compared for each of the operational modes as well as the output data. The acquisition length is the length of the data.

System mode	Spectroscopy	Relaxometry	Hysteresis loop tracing	Magnetometry	System matrix simulation
Acquisition length (ms)	83	83	83	2000	2000
Drive amplitude (mT pk)	10	10	10	1	10
Bias amplitude (mT pk)	0	0	0	50	50
Output data	Sample m(f)	Relaxation time constant	Dynamic m(H)	Quasistatic m(H)	M(f, H)

The output of this mode is the magnetization spectra as a function of frequency.

**FIG. 11.**
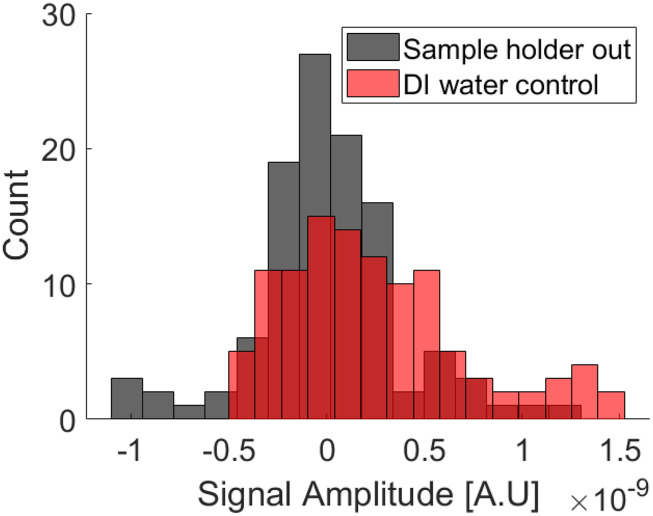
A comparative measurement of de-ionized water vs no sample (or holder) to ensure the sample holder itself was not loading the coil and biasing the results. Each count is one time-point in the in/out spectroscopy experiment. Negative signals would indicate the measured harmonic amplitude is lower with the sample in vs out. Reproduced with permission from E. Mattingly, “Design, construction, and validation of magnetic particle imaging systems for rodent, primate, and human functional neuroimaging,” Ph.D. thesis, Massachusetts Institute of Technology, 2024.^48^

To verify the water or sample holder was not causing a significant signal, we ran a sample with deionized water with identical parameters and compared it to an empty-bore dataset. Figure 11 shows the overlapping histograms for numerous measurements. Each “measurement” would be a single data point in the in/out mode time series. This indicates that the sample holder was not adding a substantial signal.

#### 4. Hysteresis loop tracing

To form the hysteresis loop of the MNP [the m(H) curve], the acquisition is the same as in a single spectroscopy measurement. The Rx data are first pre-processed as in Sec. III B 2. The drive field is measured with the BMon coil, but because the coil picks up the field’s time derivative, the signal is integrated with a cumulative trapezoidal integration function [Matlab’s “*cumtrapz*(…)”], which yields the magnetic field as a function of time.

To visualize the m(H) curve, the pre-processed Rx signal is plotted against the external field.

#### 5. Relaxometry

The magnetic domain of any real MNP will lag behind a dynamic magnetic field imposed on it due to Néel and Brownian relaxation.^53,54^ The impact of these relaxation mechanisms can be summarized by the convolution of the adiabatic magnetization [M_ad_(t)] with a relaxation kernel [r(t)],

$$M\left(t\right)=M_{ad}\;*\;r\left(t\right),$$ (7)

where M(t) is the actual magnetization response and the adiabatic, or relaxation-free, magnetization is characterized by a Langevin magnetization response,^55^

$$M_{ad}\left(t\right)=M_{sat}\cdot \left(\coth\left(\beta H\left(t\right)\right)-\frac{1}{\beta H}\right).$$ (8)

This adiabatic magnetization is convolved with a relaxation kernel; typically, the Debye kernel is used,^55–57^ which assumes a single effective time constant. For the Debye kernel, r(t) is

$$r\left(t\right)=\left(\frac{1}{\tau_{\mathrm{eff}}}e^{\frac{-t}{\tau_{\mathrm{eff}}}}\right)u\left(t\right),$$ (9)

where τ_eff_ is the effective time constant and u(t) is the Heaviside-step function.

To determine τ_eff_ for experimental data, we took in the hysteresis loop data as described earlier, and used MATLAB’s (Mathworks, Natick, MA, USA) *fminsearch*(…) function to find the best fitting time constant and Langevin parameter, *β*.

We also allowed the option for the MATLAB function to search for the best fitting two-pole relaxation kernel described by

$$r_{2}\left(t\right)=\left(\frac{1}{\tau_{1}}e^{\frac{-t}{\tau_{1}}}-\frac{1}{\tau_{2}}e^{\frac{-t}{\tau_{2}}}\right)u\left(t\right).$$ (10)

Though both of these models are profound simplifications of the underlying physics,^58,59^ the one- and two-pole models provide insights into the particle dynamics and can inform MPI reconstructions as well as particle development.

#### 6. Magnetometry

The “Magnetometry” mode’s purpose is to acquire the sample’s quasistatic magnetization as a function of an applied external field, known as the magnetization (or Langevin) curve. In this mode, it operates similarly to the superparamagnetic quantifier.^28^ This mode excites the sample with a low amplitude drive field (such that a linear approximation of the MNPs susceptibility is reasonable) and slowly biases the external DC field. By doing so, the received signal (proportional to dM/dt).

Once collected, the data from the first ∼10 bias periods are discarded to allow sufficient time for the particles to reach a periodic steady state, resulting in data that contain ∼15 remaining bias periods (∼1.5 s of data). On the cropped time series, we utilize MATLAB’s *spectrogram*(…) function with a Hanning window and a window length of 4 drive periods. The line in the spectrogram at the drive frequency is then the susceptibility as a function of time. These data are subdivided into sections based on the length of one bias period and then averaged. The susceptibility is plotted against the external bias field (measured via bias current), and this is the susceptibility curve. The integral of it is the magnetization curve, and we assume a boundary condition that the mean magnetization is zero.

#### 7. System matrix simulation

The MPI system matrix maps the vector of spectral content from the MNPs to a vector of specific locations in the imaging field of view. Inverting this linear equation provides the particle concentration density vector and, therefore, the MPI image.^10^ The system matrix is often measured by the MPI scanner by moving a point-source sample to every location within the field of view and running the acquisition scheme multiple times to ensure high SNR. Alternatively, spectrometers can be used for this purpose.^11^

To measure the 1D system matrix with a spectrometer (2D or 3D would require additional drive or bias coils), a DC field is quasi-statically superimposed on the drive field, similar to the magnetometry mode; this emulates the particles being differently biased by the gradient field in MPI. Unlike the magnetometry mode, where the particles are only lightly driven, the drive field should have the same amplitude as the imager that the system matrix corresponds to (10 mT in the example case).

The data are processed nearly identically to the magnetometry mode, except instead of only saving the line of the spectrogram corresponding to the drive frequency, the harmonics are also saved.

### C. Sensitivity performance measures

To assess the system sensitivity (minimum detectable mass, where SNR = 1 for the harmonic with the highest SNR), we utilized a ten-sample dilution series of Synomag-D 70 nm (batch = 20822104-03) ranging from 0.5 mg/ml to 7 ng/ml and made 10 *µ*l phantoms of each. The signal for each sample was plotted against its mass. The noise floor was taken as the mean noise from all of the samples in the series except the two most concentrated samples, where there was an apparent sample-correlated noise.

To determine the system’s noise floor in terms of magnetic moment in the receive coils,^34^ the system was run 50 times in three cases: (i) the preamp and drive amplifier off, showing the noise floor on the DAQ as well as any noise or interference picked up along the wires, (ii) only the receive preamp on (drive amplifier off), and (iii) the drive and receive on, but an empty bore.

The drive current stability is measured both with and without the current feedback. In either case, 500 spectroscopy measurements were taken, with each spectroscopy measurement followed by a 50 ms pause.

## IV. RESULTS

### A. Drive, receive, and bias coils

**FIG. 12.**
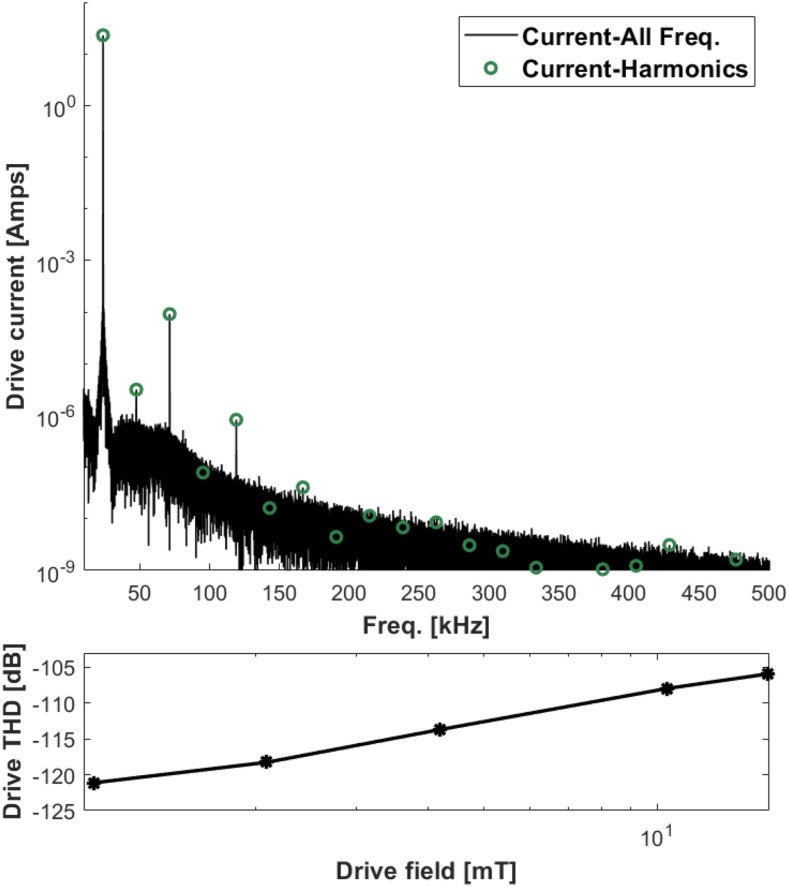
Top: Measured drive current at 10 mT field with 10 averages. The distortion is −108 dB at this amplitude. Bottom: The THD plotted as a function of drive amplitude. The maximum value tested is 15.6 mT with a distortion of −106 dB. Reproduced with permission from E. Mattingly, “Design, construction, and validation of magnetic particle imaging systems for rodent, primate, and human functional neuroimaging,” Ph.D. thesis, Massachusetts Institute of Technology, 2024.^48^

**FIG. 13.**
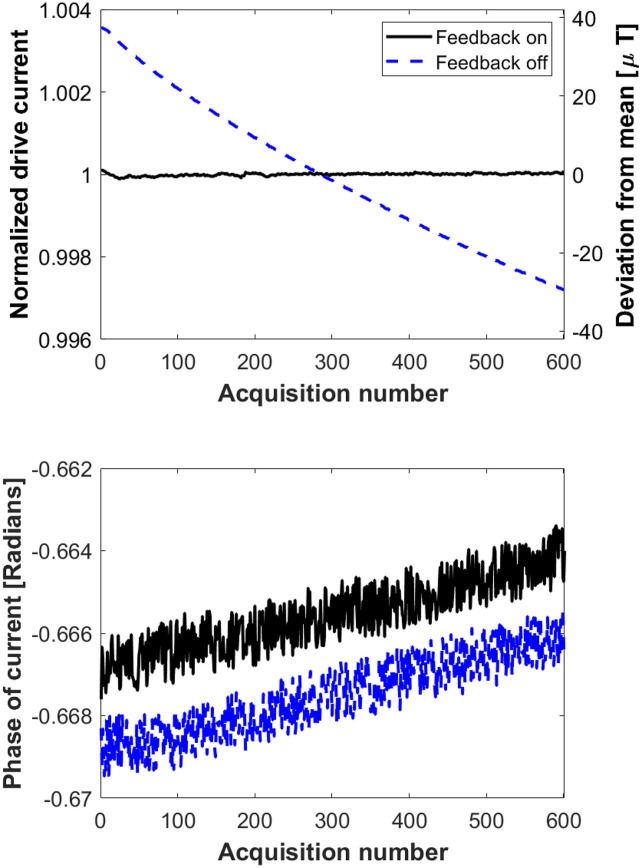
The results from the stability test of the drive coil/filter. The mean drive current was 10.5 mT for this experiment. Each point represents the current from an 84 ms measurement with a 50 ms pause between each measurement. The blue trace has no feedback to regulate the magnitude of the drive coil current, whereas the black trace has a finite impulse response network to maintain its magnitude value. Reproduced with permission from E. Mattingly, “Design, construction, and validation of magnetic particle imaging systems for rodent, primate, and human functional neuroimaging,” Ph.D. thesis, Massachusetts Institute of Technology, 2024.^48^

The drive coil meets the target 10 mT field with a 48 V/1 A supply and using a TPA3255 amplifier (Texas Instruments, Dallas, TX, USA). Using the AE Techron 7224 (AE Techron, Elkhart, IN, USA), higher amplitudes are possible, but thermal heating will limit the duty cycle. Figure 12 shows the measured drive current purity using the 7224 amplifier. The measured THD is −108 dB at 10 mT. Figure 13 shows a longer measurement at 10 mT sequence with no feedback control on the amplitude; the drift is 0.5% over 500 acquisitions (84 ms of data each) with a 50 ms pause between acquisitions. With the feedback turned on, the amplitude has minimal long-term drift.

The bias coil achieves its target field without overheating, and the receive coil does not exhibit significant skin or proximity effects up to 150 kHz, as seen in Fig. 6.

### B. System performance measures

#### 1. Spectroscopy

**FIG. 14.**
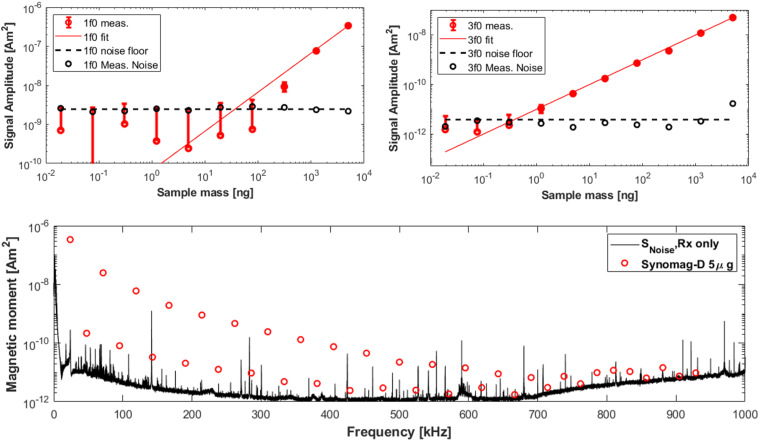
Top left: The sensitivity test results from a dilution series of Synomag-D. Each point represents one trial of “In/Out Spectroscopy” described in Sec. III B 3 for the fundamental frequency. The SNR = 1 intersection point is 37 ng. Top right: The sensitivity results for the third harmonic data. The SNR = 1 intersection is 260 pg. Bottom: The noise floor of the system resulting from the standard deviation of 50 spectroscopy runs, each run containing 84 ms of Rx data acquisition. In addition, shown is the mean value from one spectroscopy run with 5 *μ*g Fe of Synomag-D. Reproduced with permission from E. Mattingly, “Design, construction, and validation of magnetic particle imaging systems for rodent, primate, and human functional neuroimaging,” Ph.D. thesis, Massachusetts Institute of Technology, 2024.^48^

The dilution series results are seen in Fig. 14. In that experiment, the sensitivity for SNR = 1 is 37 ng for the fundamental, 260 pg for the third harmonic, and 1.0 ng for the fifth harmonic. Figure 14 also shows the measured noise (with drive at 10 mT) referred to the magnetic moment in the Rx coil. The noise at 1f_0_–5f_0_ is 107.9, 24.7, 6.7, 4.8, and 3.5 pAm^2^ (noise density of 30.1, 6.9, 1.7, 1.3, and 1.0 pAm^2^ Hz^−1/2^), respectively. When the drive coil is not powered, the noise in the second harmonic lowers to approximately the same level as the third harmonic. With the preamp turned off, the noise floor drops substantially, indicating the digitization noise is not limiting. The magnetization spectra from a 5 *μ*g sample of Synomag-D 70 nm are superimposed on the noise floor as a practical point of reference.

#### 2. Hysteresis loop tracing and relaxometry

**FIG. 15.**
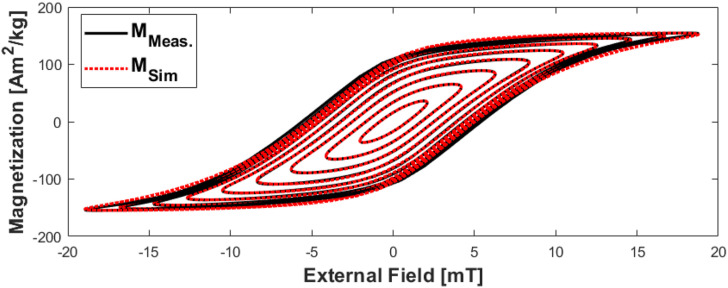
Magnetization curves for 2–18 mT with the measured data (black lines) and fitted curves (red dots). For these measurements, the two-pole model was used, and the time constants (*τ*_1_, *τ*_2_) for 2–18 mT in ascending order are [(2.78, 0.03), (2.88, 0.00), (2.88, 0.00), (2.79, 0.00), (2.04, 0.77), (1.68, 0.95), (2.32, 0.01), (2.12, 0.01), (1.95, 0.01)] *μ*s. Reproduced with permission from E. Mattingly, “Design, construction, and validation of magnetic particle imaging systems for rodent, primate, and human functional neuroimaging,” Ph.D. thesis, Massachusetts Institute of Technology, 2024.^48^

Figure 15 shows the hysteresis loops for undiluted (6 mg Fe/ml) Synomag D 70 nm samples at drive amplitudes ranging from 2 to 18 mT in steps of 2 mT. The best fit with the bi-exponential model is plotted on each measurement.

#### 3. Magnetometry

**FIG. 16.**
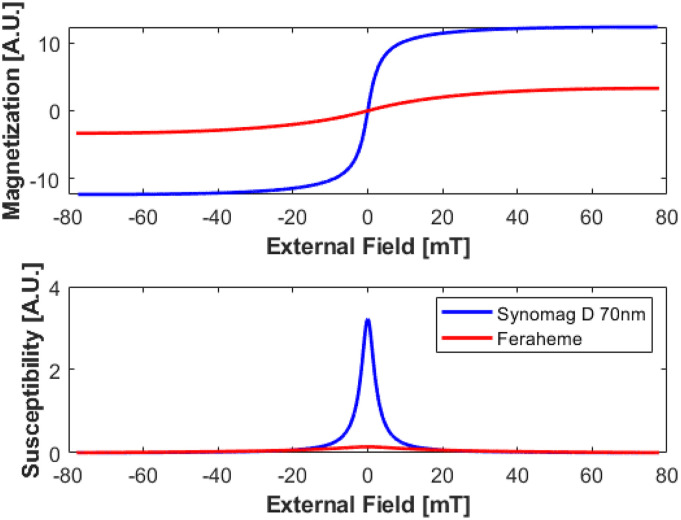
The quasistatic magnetization and susceptibility curves for Synomag-D and Feraheme normalized to the sample masses. Reproduced with permission from E. Mattingly, “Design, construction, and validation of magnetic particle imaging systems for rodent, primate, and human functional neuroimaging,” Ph.D. thesis, Massachusetts Institute of Technology, 2024.^48^

Figure 16 compares the magnetization curves for Synomag-D and Feraheme as well as the susceptibility curves. The full-width at half maximum for Synomag-D 70 nm from this measurement is 4.5 mT, and Feraheme’s FWHM is 34 mT. The saturation magnetization (normalized by mass) for Synomag-D is 3.7× higher than Feraheme.

#### 4. System matrix mode

**FIG. 17.**
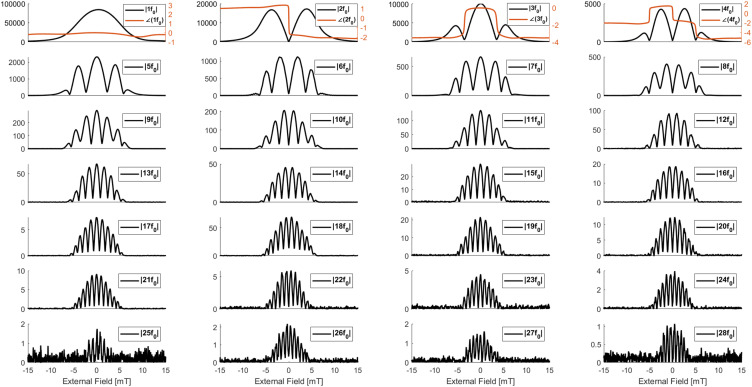
The one-dimensional “System Matrix” is the magnetic response of the sample as a function of an external field. Each subplot represents a different harmonic (1–28 are shown, up to 39th are saved). The abscissa in each is the (quasistatic) external field produced from the bias coils at about 10 Hz. The amplitude in each is the magnitude of the Fourier transform at that point in time (arb. units). The phase data are recorded for all harmonics but omitted from fifth and higher harmonics for clarity. The phases shown are unwrapped and are in radians. Reproduced with permission from E. Mattingly, “Design, construction, and validation of magnetic particle imaging systems for rodent, primate, and human functional neuroimaging,” Ph.D. thesis, Massachusetts Institute of Technology, 2024.^48^

Figure 17 shows the 1-D system matrix up to the 28th harmonic for Synomag-COOH with a 10 mT drive field amplitude. These data were acquired in 4 s (two 2 s runs averaged), and the results are consistent with the expectations of a Langevin magnetization response.

## V. DISCUSSION

The MPS device we present has the capacity to operate in five key modes: as a spectrometer outputting the harmonic spectra of magnetic nanoparticles’ internal magnetization subject to an external oscillating field (Fig. 14), plotting the MNP’s m(H) curve with its hysteresis (Fig. 15), quantifying the relaxation time constant, obtaining the quasistatic m(H) curve (Fig. 16), and measuring the one-dimensional “System Matrix” that could be used in MPI (Fig. 17).

The results from the hardware testing show we achieve the target magnetic field with an inexpensive audio amplifier (TPA3255), and higher fields are demonstrated with a more powerful amplifier (AE Techron 7224 or RCF IPS 400). The filter topology contributed to the drive field stability as well as the waveform purity. The initial target purity was −106 dBc THD, and that was slightly exceeded with a measured −108 dBc THD. While the design goal was accomplished, the overall distortion is still higher than would be expected if the only distortion source was the amplifier. This suggests component distortions play a significant role and is an avenue for future investigation, especially as drive field instability is a limiting factor for this and other MPI systems.^40^

The receive coil and preamplifier design succeeded in achieving the goal of detecting a wide dynamic range of sample masses with high sensitivity. We show results with sample masses ranging from 60 *μ*g to under 1 ng without changing the hardware configuration. The automated sample positioner is used to make multiple sample-in vs sample-out measurements. A linear model analysis then provides a particle signal measurement that is insensitive to baseline drifts. This method is a form of lock-in detection and serves to avoid the 1/f noise associated with baseline drifts. The high sensitivity and wide dynamic range are useful for a number of applications, including particle development and validation, cell counting, virus detection, thermometry, etc. A major contributing factor to the high sensitivity is the use of a step-up transformer in the receive path, which increases the signal amplitude relative to preamplifier noise. Referring to the noise on the primary side of the transformer, the voltage noise is about 0.76 nV/$\sqrt{Hz}$, which, while considerably lower than many commercial amplifiers used in MPI (e.g., SR560, Stanford Research Systems, Sunnyvale, CA, with at best 4 nV/$\sqrt{Hz}$), is still above the coil noise level. This could be improved by using discrete JFET front-ends, such as the 2SK2394 (On Semi, Scottsdale, Arizona).^33,40,50^ An improved preamp would primarily benefit the detection of higher harmonics where the drive coil instability is not the dominant noise source. Another straightforward path to improve sensitivity is to use a smaller receive coil that would fit tightly to the sample. For example, the receive coil volume is about 1 ml, and many of the samples are only 10 *µ*l.

The bias coil also satisfied the initial design requirements, and the air gaps allow for improved thermal performance. Indeed, it can easily exceed 75 mT, which is far beyond the saturation of most practical MNPs used in MPI. This enabled the characterization of Feraheme, as seen in Fig. 16.

By making the design files broadly available, we hope to provide a useful reference design that can be modified if needed. For example, a design that prioritizes drive field efficiency over maximum bias field may increase the shielding tube diameter. In this case, the bias coil’s efficiency would decrease, but the drive coil would couple less to the tube, thereby improving its performance substantially. An alternative design could use also choose to one channel of the bias amplifier for the bias field (limiting it to about half the maximum field) and the second as a drive amplifier to reduce component count and cost. Another feasible modification would be to remove the drive and receive filters, and by doing so, it would be useable as an arbitrary waveform relaxometer.^15^

## VI. CONCLUSION

We present a versatile and sensitive device capable of many different methods of particle characterization across a wide dynamic range of particle masses. The hardware is specified on a publicly available repository as part of the broader Open-source MPI project at os-mpi.github.io^60^ alongside the software to allow for replication and modifications.

## Data Availability

The data that support the findings of this study are available from the corresponding author upon reasonable request.

## APPENDIX: DRIFT SUBTRACTION PSEUDO-CODE

%% Starting with data (magnitude and phase of each harmonic, index of whether the sample was in or out, and time-stamps) in column vectors, starting with the sample out %% To clarify the pseudo-code, it has been reduced to assume data from a single harmonic, but identical processing is performed for each harmonic

1. dataComplex = dataMag * exp (1i * dataPhase)% Converting the data to be complex.
2. timeOut = time(index for sample == out)% subset of time vector when sample is out.
3. dataOut = smooth[dataComplex (index for sample == out), smoothPts]% smooth the data when the sample is out.
4. dataOut_first = mean[DataOut(1:5)]% The start of the baseline is the mean of the first five samples.
5. dataOut_last = mean[DataOut(end-4:end)]% end of the baseline is the mean of the last five samples.
6. baseline = interp1([timeOut(1) timeOut (end)], [ dataOut_first dataOut_last], timeAll, “linear,” “extrap”)% interpolate the first and last sample out points.
7. dataComplex_BaselineSub = dataComplex-baseline % data have a baseline removed.
8. dataInSmooth = smooth[dataComplex_BaselineSub(index for sample == in), smoothPts]% smooth the vector of sample-in data. smoothPts is a user input and equals 5 for the processing of the data in this paper.
9. dataOutSmooth = smooth[dataComplex_BaselineSub(index for sample == out), smoothPts]% smooth the vector of sample-out data.
10. dataComplex_BaselineSub(index for sample == in) = dataInSmooth % use smoothed data.
11. dataComplex_BaselineSub(index for sample == out) = dataOutSmooth % use smoothed data.
  %%Begin linear model processing; see Ref. 61 for additional discussion on generalized linear models and notation.
12. Y = abs(dataComplex_BaselineSub)% For remainder, only the abs is considered. Real and imaginary components can be processed similarly, or the data can be processed as complex. Context will dictate which is appropriate.
13. constant = ones[size(dataToProc)]% A constant vector.
14. linear = time/time(end)% Making a ramp from 0 to 1.
15. InOut = index for sample == in; % Making a vector where 1 = sample in, 0 = sample out.
16. X = [InOut(:), const(:), linear(:)]% Making a matrix of regressors, additional regressors such as quadratic or cubic terms could be added.
17. Betas = inv(X’ * X) * X’ * Y; % Betas gives the contribution of each regressor to the net signal, so Betas(1) is the amplitude of the in vs out component of the time-series.
18. Yhat = X * Betas; % YHat is the reconstructed time-series given the regressors (includes drift terms).
19. nHat = Y-Yhat; % nHat is the error.
